# Effects of Shiga Toxin Type 2 on Maternal and Fetal Status in Rats in the Early Stage of Pregnancy

**DOI:** 10.1155/2014/384645

**Published:** 2014-05-05

**Authors:** Flavia Sacerdoti, María M. Amaral, Elsa Zotta, Ana M. Franchi, Cristina Ibarra

**Affiliations:** ^1^Laboratorio de Fisiopatogenia, Departamento de Fisiología, Instituto de Fisiología y Biofísica Bernardo Houssay (IFIBIO Houssay-CONICET), Facultad de Medicina, Universidad de Buenos Aires, 2155 Paraguay street, Piso 6, 1121 Buenos Aires, Argentina; ^2^CEFYBO-CONICET, Universidad de Buenos Aires, 2155 Paraguay Street, Piso 16, 1121 Buenos Aires, Argentina

## Abstract

Shiga toxin type 2 (Stx2), a toxin secreted by Shiga toxin-producing *Escherichia coli* (STEC), could be one of the causes of maternal and fetal morbimortality not yet investigated. In this study, we examined the effects of Stx2 in rats in the early stage of pregnancy. Sprague-Dawley pregnant rats were intraperitoneally (i.p.) injected with sublethal doses of Stx2, 0.25 and 0.5 ng Stx2/g of body weight (bwt), at day 8 of gestation (early postimplantation period of gestation). Maternal weight loss and food and water intake were analyzed after Stx2 injection. Another group of rats were euthanized and uteri were collected at different times to evaluate fetal status. Immunolocalization of Stx2 in uterus and maternal kidneys was analyzed by immunohistochemistry. The presence of Stx2 receptor (globotriaosylceramide, Gb3) in the uteroplacental unit was observed by thin layer chromatography (TLC). Sublethal doses of Stx2 in rats caused maternal weight loss and pregnancy loss. Stx2 and Gb3 receptor were localized in decidual tissues. Stx2 was also immunolocalized in renal tissues. Our results demonstrate that Stx2 leads to pregnancy loss and maternal morbidity in rats in the early stage of pregnancy. This study highlights the possibility of human pregnancy loss and maternal morbidity mediated by Stx2.

## 1. Introduction

Maternal bacterial and viral infections during pregnancy are associated with an increased incidence of fetal death, congenital malformations, placental abruption, premature rupture of membranes, and prematurity in humans and animals [1]. Spontaneous miscarriage is the most common adverse pregnancy outcome in humans and occurs in 15–20% of all recognized pregnancies. Early pregnancy loss is a common complication of human pregnancy and the causes are often unknown and unstudied. The causes for a spontaneous miscarriage are diverse and comprise genetic, endocrinologic, anatomic, immunologic, or microbiologic aspects [2]. Although miscarriage represents the most common complication in pregnancy and as such has been thoroughly investigated, the causes of miscarriages are still unexplained.

In the majority of cases [3], it is well known that the pregnant state is associated with increased sensitivity to endotoxin in renal and uterine circulation [4] and maternal infection could cause abortion and preterm labour in women [5]. Infections are accepted as a cause of late fetal demise and could be responsible for early fetal losses as well [3].

Shiga toxin-producing* Escherichia coli *(STEC) are gastrointestinal bacteria whose infection can cause diarrhea and hemorrhagic colitis. The systemic complication of STEC infection is known as hemolytic uremic syndrome (HUS), caused principally by the effects of Shiga toxin (Stx) in target organs like kidney and brain. STEC infection is mostly seen in young children including neonates [6, 7] although the outbreak in 2011 in central Europe caused by Stx2-producing STEC affected more adults than children, and women were overrepresented [8–10]. STEC have a zoonotic origin and they can be transmitted directly or indirectly from animals to humans. STEC are present in the intestinal tract of healthy cattle, and transmission occurs not only by consumption of undercooked ground beef, manure contaminated water, vegetables, and fruit, and unpasteurized milk, but also by direct contact from person to person [11]. After the ingestion of bacteria, STEC colonize the human intestine where Stx is released and crosses the intestinal mucosal barrier. Then, toxin circulates in the bloodstream and reaches its target organs, mainly kidney and brain [12]. Stx has an AB_5_ molecular configuration where an enzymatically active monomeric A subunit is noncovalently associated with a pentamer of B subunit which is responsible for binding to globotriaosylceramide (Gb3) (this receptor can be also named CD77 or P_*k*_ blood group antigen) receptor [13]. Gb3 is located on the plasma membrane of target cells, particularly endothelial cells present in kidneys, brain, and other organs [14, 15]. Binding of Stx to Gb3 is the primary determinant of its cytotoxic effects and results in toxin internalization and cell killing by inhibition of protein synthesis and induction of apoptosis [16]. To our knowledge, there are no reports of Stx2 effects during human pregnancy or described complications in the early pregnancy associated to STEC infection. However, several cases of hemolytic uremic syndrome (HUS) after STEC infections in postpartum women and neonate have been reported [6, 17–19]. Also STEC, serotype O15:Krvc383, was diagnosed as the putative cause of placentitis and late-gestation abortion in ewes [20]. The hypothesis is that early pregnancy can be affected by Stx2 through a direct effect in the uteroplacental unit. This event could trigger complications or pregnancy loss. The purpose of the present study was to examine the cytotoxic effects of Stx2 in rats in the early stage of pregnancy after the event of implantation. We propose that the systemic circulation of Stx2 in the maternal vessels can affect the highly irrigated uteroplacental unit and induce fetal and placental responses associated with fetoplacental injury, leading to abortion or pregnancy complications. The rationale for this study is to evaluate the possibility of Stx2-mediated pregnancy complications not previously considered and to direct further attention to the role of soluble products of microorganisms in the pathogenesis of abnormal gestation and pregnancy complications.

## 2. Materials and Methods

### 2.1. Drugs and Chemicals

Purified Stx2 was purchased from Phoenix Laboratory, Tufts Medical Center, Boston, MA, USA, and it was checked for lipopolysaccharide (LPS) contamination by* Limulus amoebocyte *lysate assay (Biowhittaker Inc., MD, USA). Toxin was diluted with sterile phosphate-buffered saline (PBS) before injection. Stx2 contained <10 pg LPS/ng of pure Stx2. Gb3 standard was purchased from Matreya (Pleasant Gap, PA, USA).

### 2.2. Animals

Timed pregnant rats were obtained mating male and virgin female Sprague-Dawley rats (200–280 g; 2-3 months of age), acquired from the Animal Facility of the School of Pharmacy and Biochemistry. Mating was performed placing female rats in the cages of the male rats from the same strain for several days. Day 1 of gestation was determined when sperm was observed in the vaginal smear. The animals received food and water* ad libitum *and were housed under controlled conditions of light (12 h light; 12 h dark) and temperature (23–25°C). This study was carried out in strict accordance with the recommendations detailed in the Guide for the Care and Use of Laboratory Animals of the National Institutes of Health. Protocols were approved by the Committee for the Care and Use of Laboratory Animals of the University of Buenos Aires (CICUAL, Permit Number 2954/10 and 1494/2013). Three to six animals were used in each experimental group. The experiments were repeated at least twice.

### 2.3. Evaluation of Maternal Status

In order to examine the effects of Stx2 on maternal status, pregnant rats on day 8 of gestation (gd 8; day of delivery = 22-23 days) were randomly divided into three groups of 4 animals each. This experiment was repeated twice. One group was injected with a single dose of 0.25 ng Stx2/g bwt, and the other group was treated with a single dose of 0.5 ng Stx2/g bwt. Control rats were injected with the same volume (1 *μ*L/g bwt) of PBS. Pregnant rats were individually housed, weighted, and checked for water (mL) and food intake (g) every 24 h, until 9 days after injection. Rats were followed until delivery to evaluate delivery time. Signs of illness like piloerection, inactivity, and vaginal bleeding were analyzed. Data of weight are presented as Δweight (body weight at *n* day after injection-body weight at 0 days after injection).

### 2.4. Evaluation of Fetal Status

After the evaluation of the maternal status and progression of pregnancy, other groups of rats of 3–6 animals each were injected with both doses of Stx2 and PBS. Rats were sacrificed at 10 days after injection to evaluate fetal status and the number of resorptions. Uteri were removed and total fetal resorptions were macroscopically evaluated. The experiment was repeated twice.

### 2.5. Gross Evaluation of the Uteroplacental Unit

For gross evaluation of the fetal status after injection of both Stx2 doses and PBS, groups of 3–6 animals each were sacrificed at 2, 4, and 6 days after injection. Uteri were removed and the status of the uteroplacental units was macroscopically evaluated. Maternal kidneys and uteri were then formalin fixed for histological evaluation. The experiment was repeated twice.

### 2.6. Histology

Uteroplacental units and maternal kidneys from rats at 6 days after injection were fixed for 48 h with formalin 10% in PBS 0.1 M (pH 7.4). Two or three uteroplacental units from every uterus (groups of 3–6 animals each) were randomly dissected, dehydrated, and embedded in paraffin. Sections of 5 *μ*m were made by a microtome (Leica RM 2125, Wetzlar, Germany) and mounted on 2% silane coated slides. The sections were stained with hematoxylin and eosin (H&E) and observed by light microscopy (Nikon Eclipse 200, NY, USA).

### 2.7. Stx2 Immunohistochemistry

For Stx2 detection in maternal kidney and uteroplacental units, animals injected with 0.5 ng/g bwt (total body weight) and controls were euthanized after 6 h of treatment and finally formalin fixed for immunohistochemical studies.

Briefly, tissues were fixed with formalin 10% in PBS at room temperature. The samples were then dehydrated and embedded in paraffin. Tissue sections of 5 *μ*m thickness were deparaffinized and hydrated, and subsequently endogenous peroxidase was blocked with hydrogen peroxide (H_2_O_2_) 3% in PBS for 20 min and rinsed with PBS. Later, the slides were blocked with a 5% solution of dry milk in PBS in a humidity chamber at room temperature for 90 min to prevent nonspecific protein binding. Sections were incubated overnight at 4°C with the mouse monoclonal antibody 2E11 (1 : 20; 12.5 *μ*g/mL) (kindly provided by Dr. Roxane M. F. Piazza) against the A-subunit of Stx2 [21]. Sections were then washed with PBS and incubated for 90 min at room temperature with a rabbit anti-mouse (Sigma-Aldrich Co., 1 : 100) conjugated with peroxidase enzyme. Finally, slides were incubated with 3,3′-diaminobenzidine (DAB, Sigma-Aldrich Co.) and H_2_O_2_, counterstained, dehydrated, and mounted for observation. An isotype control was performed with a mouse monoclonal anti-BrdU, dilution 1 : 20 (clone DU 33 #B 2531, Sigma, St. Louis, MO, USA), where no staining was detected, not in decidua nor in the kidney (data not shown). This assay was repeated at least twice.

### 2.8. Gb3 Determination

To assess whether Stx2 could bind to decidua and uterus from pregnant rats at 8 days of gestation, Gb3 presence in uterus and decidua was investigated by thin layer chromatography (TLC). Neutral glycolipids from decidua and uterus from rats of 8 days of gestation were extracted according to the method of Bligh and Dyer with minor modifications [22]. Briefly, 100 mg of tissue was weighted and homogenized on ice with an Ultra-Turrax homogenizer with 0.8 mL of HES buffer (10 mM HEPES KOH, 0.1 mM of EDTA, and 250 mM of sucrose). Three mL of chloroform : methanol 2 : 1 v/v was added to the homogenate, vortexed for 30 s, and incubated on ice for 15 min. Two mL of chloroform : water 1 : 1 v/v was added to the tube and centrifuged at 3000 rpm for 5 min to separate phases. The bottom phase was recovered and evaporated to dryness. One mL of methanol and 0.1 mL of 1.0 M NaOH were added to the dried residue and incubated at 37°C overnight. Two mL of chloroform and 0.5 mL of water were added to the tube, vortexed for 30 s, and centrifuged for 5 min at 3000 rpm. The lower phase corresponding to the neutral glycolipids was brought to dryness. The residue was then suspended in 40 *μ*L of chloroform: methanol 2 : 1 v/v and applied to the bottom of the silica TLC plate (Merck Química Argentina). The chromatography was developed in a glass tank with a mixture of chloroform: methanol: water (60: 35: 8). A purified Gb3 standard (Matreya, Pleasant Gap, PA, USA) was also added to the plate for comparison. After the solvent front reaches the top of the plate, the gel matrix was air-dried and treated with a solution of orcinol (50 mg orcinol, 10 mL of sulfuric acid, and 39.5 mL of water) to visualize the glycolipids.

### 2.9. Creatinine Determination

For creatinine determination, blood was collected by cardiac puncture of CO_2_ anesthetized rats injected with 0.5 ng Stx2/g bwt and PBS at 2, 4, and 6 days after injection (groups of 3–6 animals each). Plasma creatinine concentration was determined using a commercial kit (Wiener Lab, Argentina) according to the manufacturer's recommendations. The experiment was repeated twice.

### 2.10. Statistical Analysis

Statistical analysis was performed using the Graph Pad Prism Software 5.0 (San Diego, CA, USA). ANOVA for repeated measures was used to calculate differences between groups in weight and food and water intake and Tukey's multiple comparisons test was used as* a posteriori *test. Differences in the number of fetuses and fetal resorptions were analyzed by Kruskal-Wallis test, using Dunn's multiple comparisons test as* a posteriori *test. Differences in plasma creatinine concentration and delivery of pups between Stx2-treated rats and controls were analyzed by Mann-Whitney test. Statistical significance was set at *P* < 0.05.

## 3. Results

### 3.1. Progression of Pregnancy in Rats Treated with Stx2

**Figure 1:**
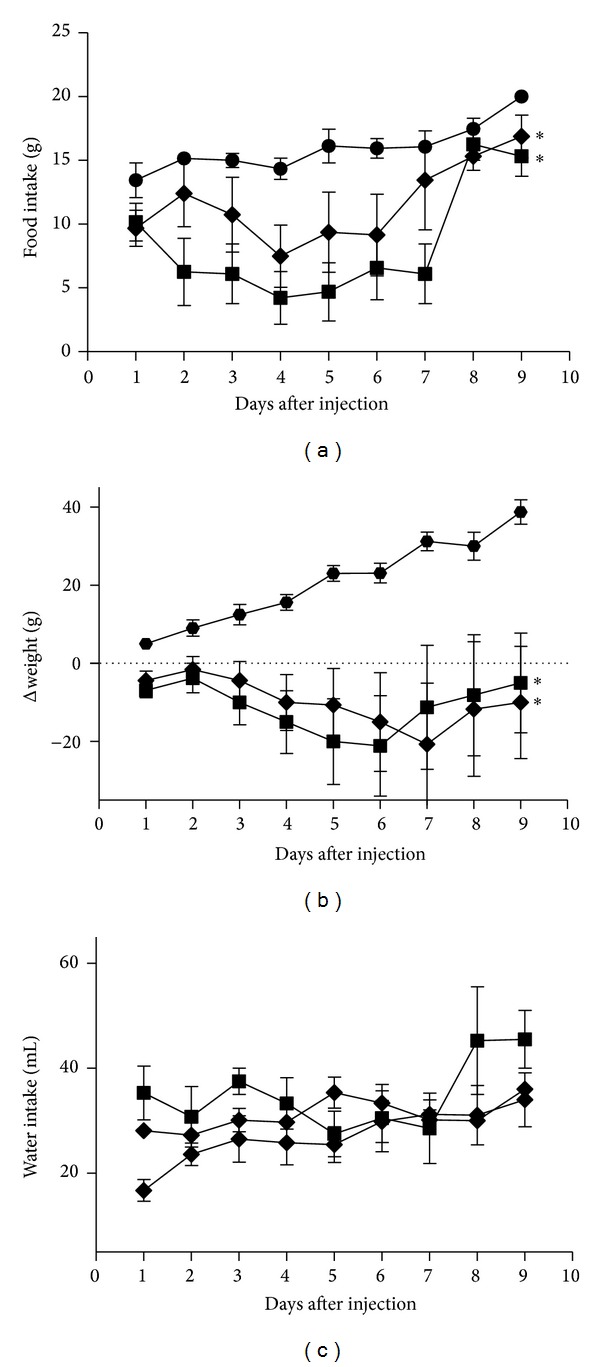
Evaluation of body weight and food and water intake. Time course of food intake (a), body weight (b), and water intake (c) of pregnant rats i.p. injected with PBS (Ctrl, -●-), 0.25 ng Stx2/g bwt (-◆-), or 0.5 ng Stx2/g bwt (-■-). Each point of the curves represents the mean ± SEM. In (a) and (b), *P < 0.05 for 0.25 (*n* = 8) and 0.5 ng Stx2/g bwt (*n* = 8) versus Ctrl (*n* = 8). In (c), there was no significant difference between groups.

Doses of 0.25 and 0.5 ng Stx2/g bwt i.p. injected into pregnant rats caused signs of illness like piloerection and inactivity from 2 days after injection until 7-8 days after injection. Furthermore, a progressive decrease of food intake until day 7 after injection was observed (Figure 1(a)) with a consequent fall of weight compared to controls (Figure 1(b)). Seven-eight days after injection, rats gradually recovered appetite and weight, although they did not reach the weight corresponding to the time of pregnancy. All rats injected with 0.25 and 0.5 ng Stx2/g bwt showed vaginal bleeding on days 6–10 after injection. Controls gradually gained weight as expected (Figure 1(b)). There were no differences in water intake in Stx2-treated rats compared with their controls (Figure 1(c)). Rats treated with the dose of 0.5 ng Stx2/g did not deliver any pups, while a few rats treated with the dose of 0.25 ng Stx2/g delivered some normal pups at term 6 ± 2 versus control 13 ± 2 (*P* < 0.05, *n* = 5).

### 3.2. Effect of Stx2 on Fetal Status at 10 Days after Injection

The number of fetoplacental resorptions in rats injected with 0.5 ng Stx2/g bwt was significantly higher than that observed in control rats (Table 1). There was also a significantly higher fetoplacental resorption in rats injected with 0.25 ng Stx2/g bwt compared with controls.

### 3.3. Structural Alterations in the Uteroplacental Unit

**Figure 2:**
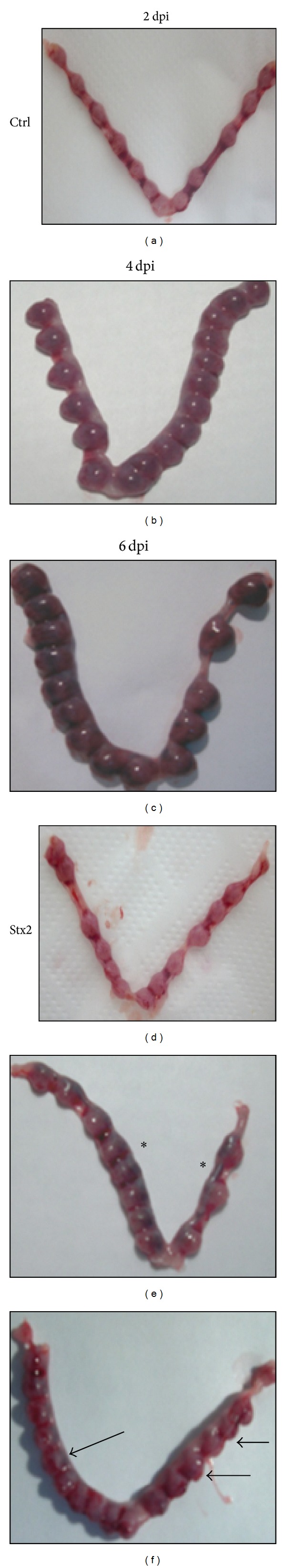
Gross evaluation of pregnant uterus. Pregnant rats were injected with PBS or 0.5 ng Stx2/g bwt at different times. No differences were observed at 2 days after injection (d) compared to control (a). Growth restriction and hemorrhage were observed in uteroplacental units injected with 0.5 ng Stx2/g bwt at 4 days after injection (asterisk (e)), and fetal resorptions were observed at 6 days after injection (black arrows (f)) compared with controls ((b) and (c), resp.).

Structural alterations in the uteroplacental unit were analyzed at 2, 4, and 6 days after injection. No differences were observed at 2 days after injection (Figure 2(d)) with respect to controls (Figure 2(a)). Growth restriction and hemorrhage were observed in uteroplacental units injected with 0.5 ng/g bwt at 4 days after injection (Figure 2(e)) and fetal resorptions were observed at 6 days after injection (Figure 2(f)) compared with controls (Figures 2(b) and 2(c), resp.). Macroscopical alterations were observed with 0.25 ng/g bwt dose but with less damage compared with the other dose (data not shown).

**Figure 3:**
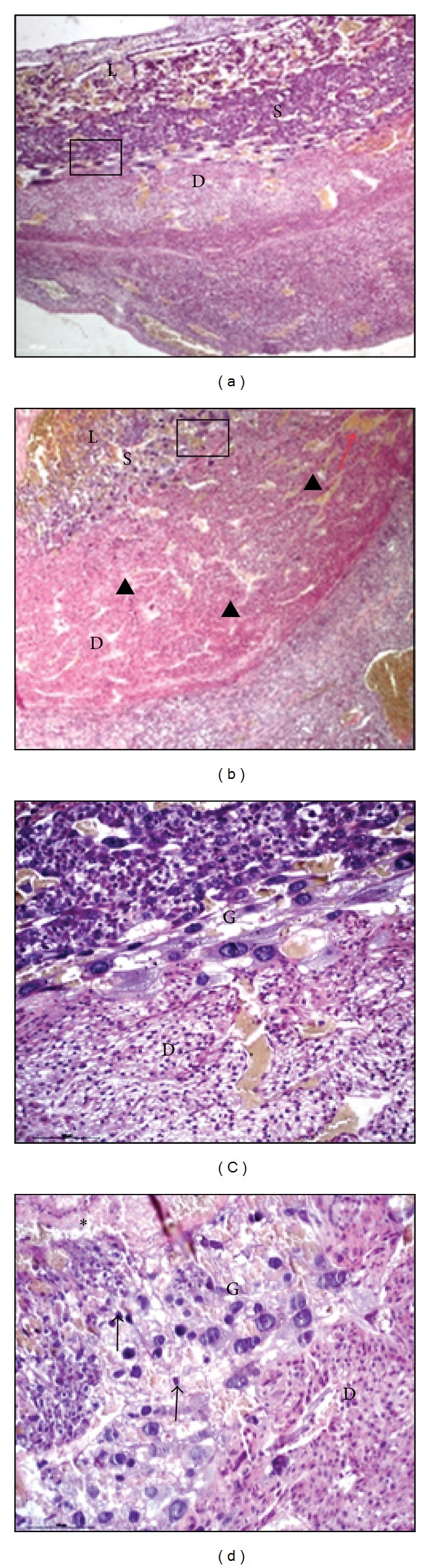
Histological evaluation of the uteroplacental unit.**  **Alterations in the histoarchitecture (black arrowheads (b)) and isolated focus of hemorrhage (red arrow (b)) were observed in Stx2-treated rats. High magnification showed trophoblast fragmented nuclei (black arrows (d)) and necrosis area (black asterisk (d)). Normal tissues were observed in control rats ((a) and (c)). Black squares in (a) and (b) indicate the magnification zone corresponding to (c) and (d), respectively. (a, b): ×40; (c, d): ×200. D, decidua; G, trophoblast giant cells; L, labyrinth; S, spongious layer.

**Figure 4:**
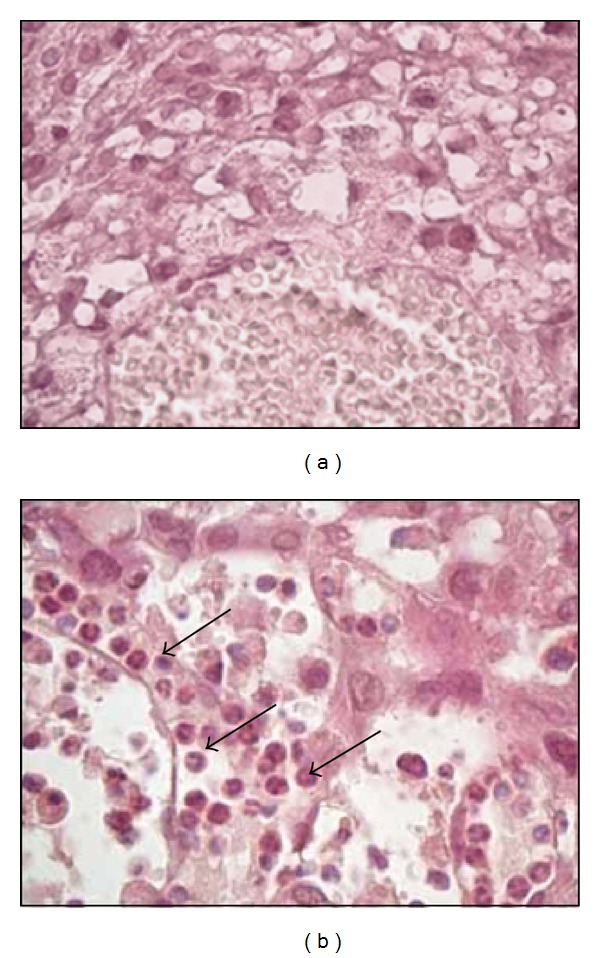
Leukocyte infiltration in decidua. Leukocyte infiltration in decidua tissues from the Stx2-treated rats (0.5 ng/g bwt) was observed at 4 days after injection (black arrows (b)). Decidua tissues from control rats did not show leukocyte infiltration (a).

For histological evaluation, uteroplacental units were collected at 4 and 6 days after injection of rats with 0.5 ng Stx2/g bwt. Alterations in the placental histoarchitecture with isolated haemorrhage (Figure 3(b)), necrotic areas, and trophoblast fragmented nuclei (Figure 3(d)) were observed in Stx2-treated rats compared with controls (Figures 3(a) and 3(c), resp.). Leukocyte infiltration in decidua was also observed in Stx2-treated rats (Figure 4(b)), but it was absent in control rats deciduas (Figure 4(a)).

### 3.4. Detection of Gb3 and Stx2 in Decidua Tissues

**Figure 5:**
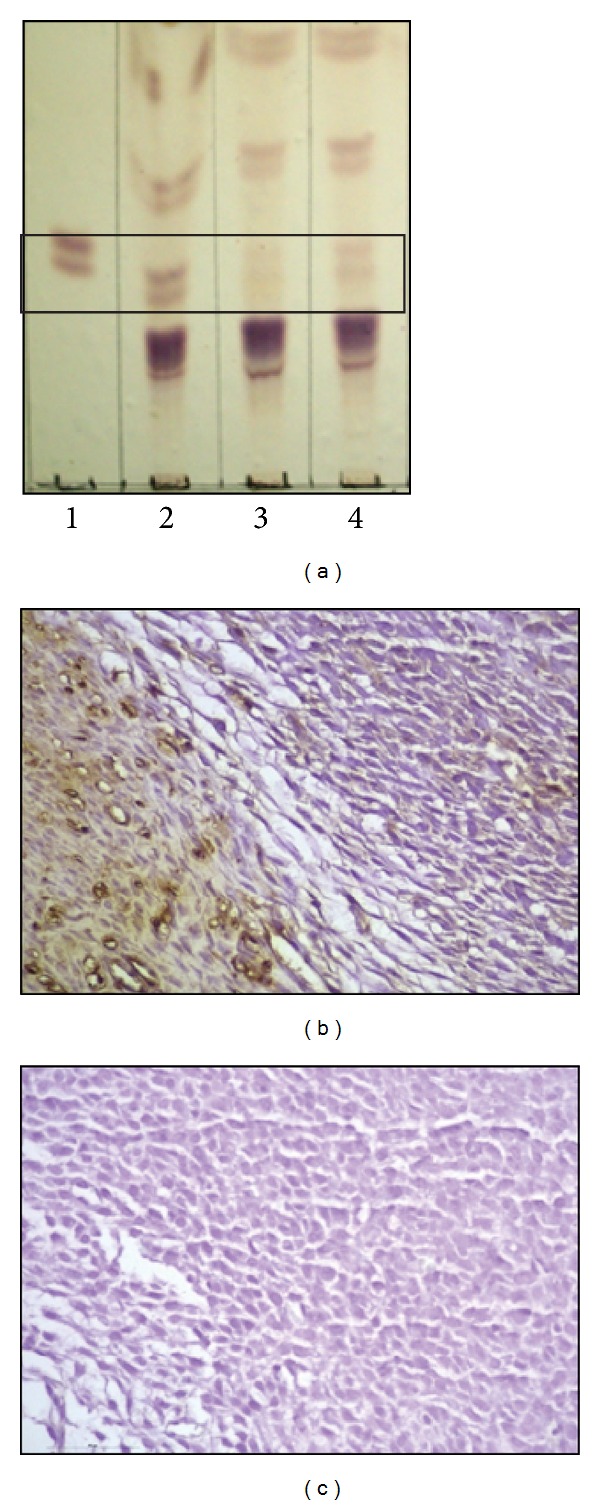
Detection of Gb3 and Stx2 in decidua tissues. Gb3 was detected in decidua tissues from normal pregnant rats at day 8 of gestation by TLC ((a), line 1: standard of Gb3; line 2: decidual and embryo cells; line 3: uterus; line 4: complete site of implantation including uterus decidua and embryo cells). Stx2 was detected in the microvasculature and decidua cells of the uteroplacental unit at 6 h after injection with 0.5 ng Stx2/g bwt (b). No staining was detected in control tissue (c). (b, c): ×400.

To determine if Gb3 (Stx2 receptor) is present in the uteroplacental unit, decidua and uterus tissues from normal rats at gd 8 were subjected to lipid extraction followed by TLC (Figure 5(a)). Two bands located at the same distance of Gb3 standard revealed the presence of Gb3 in decidua and embryo cells but not in the rat uterus. Furthermore, Stx2 was immunolocalized in the microvasculature and decidua cells of Stx2-treated rats at 6 h after injection (Figure 5(b)). There was no staining in decidua from control rats (Figure 5(c)).

### 3.5. Structural and Functional Alterations in Maternal Kidneys

**Figure 6:**
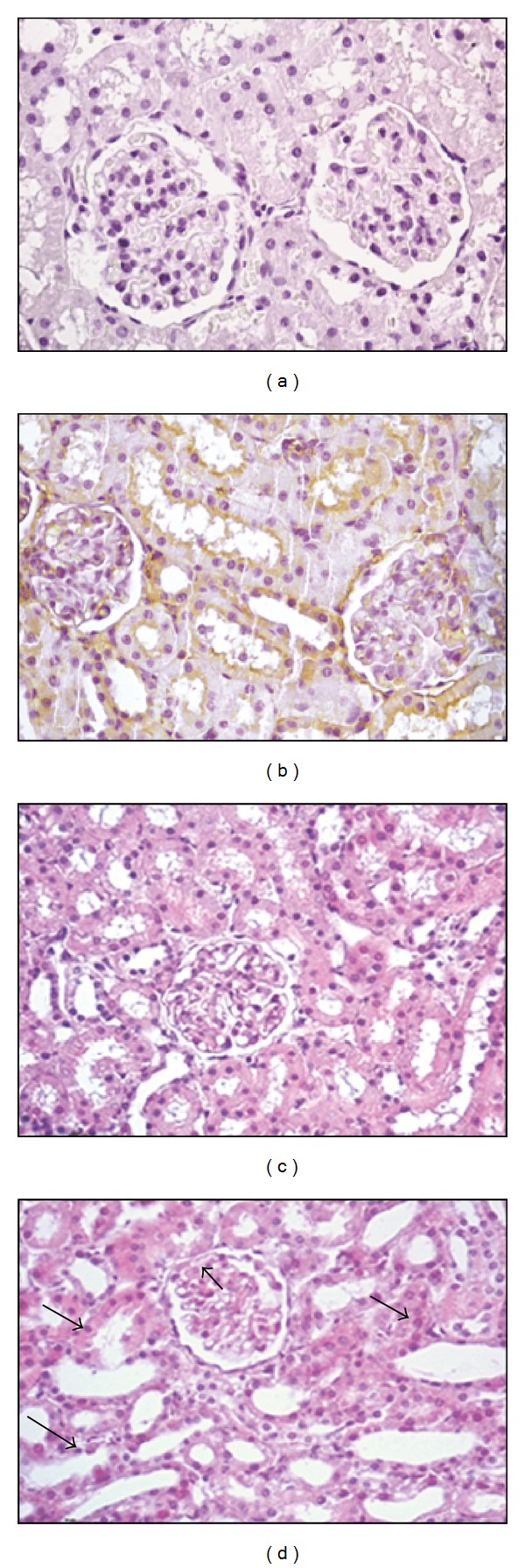
Stx2 localization in maternal kidney and histological evaluation. Stx2 was detected in glomerular and tubular cells of 0.5 ng Stx2/g bwt treated rats at 6 h after injection by immunohistochemistry (b). This assay was repeated at least twice. No staining was detected in control renal tissues (a). Glomerular epithelial adherences (black arrowhead) and tubular necrosis (black arrows) were observed in Stx2-treated rats at 6 days after injection (d) compared with the controls (c). (a, b): ×400; (c, d): ×200.

To further analyze histological changes in kidneys due to Stx2, renal tissues from pregnant rats treated with 0.5 ng Stx2/g bwt at 6 days after injection were processed and stained with H&E. Glomerular epithelial adherences and tubular necrosis were detected, by light microscopy, in Stx2-treated rats (Figure 6(d)) compared with controls (Figure 6(c)). Stx2 was detected by immunohistochemical staining in glomerular and tubular cells at a short time of 6 h after injection (Figure 6(b)). No staining was detected in control renal tissues (Figure 6(a)). Furthermore, a moderate increase in creatinine plasma concentration (mg/dL) was observed in rats treated with 0.5 ng Stx2/g bwt at 4 days and 6 days after injection compared with controls (Table 2). In contrast, no significant changes in creatinine plasma concentration were detected in rats treated with 0.25 ng Stx2/g bwt. In addition, at 2 days after injection, there were no differences in creatinine concentrations with both doses compared to controls (Table 2).

## 4. Discussion

In this study, we have evaluated the effects of sublethal Stx2 doses on maternal and fetal status in the early postimplantation stage of pregnancy (gd 8). An i.p. injection of 0.25 and 0.5 ng/g bwt of Stx2 caused maternal damages and pregnancy loss. Maternal infections have been described as cause of spontaneous abortion and perinatal complications. We propose the possibility that high circulation of STEC strains could trigger pregnancy complications due to the release of virulence factors like Stx2 in symptomatic or asymptomatic STEC infections. Although a case of neonatal HUS after mother-to-child transmission of STEC O157:H7 has been reported [6, 18, 23], up to date, no data is available regarding loss of pregnancy linked to exposure to STEC in humans.

We have previously reported that a combination of Stx2 and LPS i.p. injected in rats in the late stage of pregnancy produces preterm delivery of dead fetuses [24]. We also demonstrated that nitric oxide (NO) and tumor necrosis factor (TNF-*α*) play an important role in placental toxicity and fetal mortality induced by Stx2 [24–26]. In addition, it has been reported that STEC cause placentitis and abortion in ewes [20].

In this study, we have demonstrated that sublethal Stx2 doses in the early stage of pregnancy induced maternal weight loss and a decrease in food intake until 7 days after injection. After that, rats gradually recovered, as shown in several models of Stx2 injection [27], but did not reach the appropriate weight for the corresponding day of pregnancy. Moreover, our results indicated that Stx2 was localized in the microvasculature and decidua cells where high levels of Gb3 were found. In agreement with our results, Yoshimura et al. [28] reported that Stx2 induces fetoplacental resorption in mice in the early stage of pregnancy [28]. These authors also found that Stx2 injures the trophoblasts, causing intrauterine hemorrhage, fibrin deposition, and neutrophil infiltration. In accordance with this, our experiments demonstrated significant morphological and histological damages in the uteroplacental units and fetomaternal resorptions at 6–10 days after injection of Stx2. We have detected leukocyte infiltration in decidua from Stx2-treated rats, which could be the result of transmigration from the blood microvasculature to decidua tissues due to endothelial damages caused by Stx2 [29].

In this study, we have found histological and physiological renal alterations in pregnant rats depending on the Stx2 dose. It is known that kidneys develop physiological adaptations to transition through gestation [30] and therefore the combination of kidney disease and pregnancy could be a high-risk situation in humans [31]. However, a 5/6 reduction of renal mass in pregnant rats does not alter glomerular hemodynamic responses to pregnancy [32]. Furthermore, in the data presented here, loss of pregnancy at 0.25 ng Stx2/g bwt without functional kidney alterations was observed. Therefore, the possibility that maternal renal damages contribute to Stx2-pregnancy loss is unlikely.

In conclusion, we propose that the detrimental effects on pregnancy are related to the direct cytotoxic effects of Stx2 in the highly blood perfused uteroplacental unit. It is well known that Stx2 affects the endothelium of microvasculature [14, 33], and, in this work, we found Stx2 binding the microvasculature of the uteroplacental unit. Our data suggest that these Stx2 cytotoxic effects could be responsible for intrauterine growth restriction and final pregnancy loss.

Even though we cannot directly extrapolate our results to the human placental response, it is tempting to propose that Stx2 exposure during pregnancy could be investigated as one of the causes of spontaneous miscarriage.

It is well known that rodents still possess significant similarities with humans, in terms of reproductive biology [34], so the fact that Stx2 causes pregnancy loss and preterm delivery in rats may be an indication that this event could happen in humans.

## Figures and Tables

**Table 1:** Effect of Stx2 on fetal status at 10 days after injection.

Stx2 dose	Number of rats	Number of fetuses	Number of resorptions
Control	10	13 ± 1	1 ± 1
0.25 ng/g bwt	6	4 ± 2*	8 ± 2*
0.5 ng/g bwt	12	1 ± 1**	13 ± 1**

*P < 0.05 and **P < 0.001 versus control.

**Table 2:** Plasma creatinine in Stx2-treated rats.

Stx2 dose	Number of rats	Plasma creatinine, mg/dL; days after Stx2 injection		
		2	4	6
Control	8	0.53 ± 0.03	0.48 ± 0.03	0.53 ± 0.05
0.25 ng/g bwt	5	0.65 ± 0.12	0.65 ± 0.11	0.55 ± 0.05
0.5 ng/g bwt	6	0.63 ± 0.03	0.81 ± 0.14*	0.85 ± 0.15*

*P < 0.05 versus control.

## References

[1] Gasic GJ, Gasic TB, Strauss JF (1975). Abortifacient effects of *Vibrio cholerae* exo enterotoxin and endotoxin in mice. *Journal of Reproduction and Fertility*.

[2] Nepomnaschy PA, Sheiner E, Mastorakos G, Arck PC (2007). Stress, immune function, and women's reproduction. *Annals of the New York Academy of Sciences*.

[3] Matovina M, Husnjak K, Milutin N, Ciglar S, Grce M (2004). Possible role of bacterial and viral infections in miscarriages. *Fertility and Sterility*.

[4] Cipolla MJ, Houston EM, Kraig RP, Bonney EA (2011). Differential effects of low-dose endotoxin on the cerebral circulation during pregnancy. *Reproductive Sciences*.

[5] Lamont RF, Sawant SR (2005). Infection in the prediction and antibiotics in the prevention of spontaneous preterm labour and preterm birth. *Minerva Ginecologica*.

[6] Ulinski T, Lervat C, Ranchin B, Gillet Y, Floret D, Cochat P (2005). Neonatal hemolytic uremic syndrome after mother-to-child transmission of *Escherichia coli* O157. *Pediatric Nephrology*.

[7] Flandrois M, Bessière A, Vieira-Roth S, Vergnaud M, Frémeaux-Bacchi V, Eckart P (2011). Hemolytic and uremic syndrome and maternal-fetal *Escherichia coli* K1 infection. *Archives de Pediatrie*.

[8] Rasko DA, Webster DR, Sahl JW (2011). Origins of the *E. coli* strain causing an outbreak of hemolytic-uremic syndrome in Germany. *The New England Journal of Medicine*.

[9] Bielaszewska M, Idelevich EA, Zhang W (2012). Effects of antibiotics on Shiga toxin 2 production and bacteriophage induction by epidemic *Escherichia coli* O104:H4 strain. *Antimicrobial Agents and Chemotherapy*.

[10] Frank C, Werber D, Cramer JP (2011). Epidemic profile of Shiga-toxin-producing *Escherichia coli* O104:H4 outbreak in Germany. *The New England Journal of Medicine*.

[11] Hussein HS (2007). Prevalence and pathogenicity of Shiga toxin-producing *Escherichia coli* in beef cattle and their products. *Journal of Animal Science*.

[12] Ibarra C, Amaral MM, Palermo MS (2013). Advances in pathogenesis and therapy of hemolytic uremic syndrome caused by Shiga toxin-2. *IUBMB Life*.

[13] Waddell T, Cohen A, Lingwood CA (1990). Induction of verotoxin sensitivity in receptor-deficient cell lines using the receptor glycolipid globotriosylceramide. *Proceedings of the National Academy of Sciences of the United States of America*.

[14] Obrig TG, Louise CB, Lingwood CA, Boyd B, Barley-Maloney L, Daniel TO (1993). Endothelial heterogeneity in Shiga toxin receptors and responses. *The Journal of Biological Chemistry*.

[15] Müthing J, Schweppe CH, Karch H, Friedrich AW (2009). Shiga toxins, glycosphingolipid diversity, and endothelial cell injury. *Thrombosis and Haemostasis*.

[16] Bergan J, Lingelem ABD, Simm R, Skotland T, Sandvig K (2012). Shiga toxins. *Toxicon*.

[17] Steele BT, Goldie J, Alexopoulou I, Shimizu A (1984). Post-partum haemolytic-uremic syndrome and verotoxin-producing *Escherichia coli*. *The Lancet*.

[18] Lienemann T, Salo E, Rimhanen-Finne R (2012). Shiga toxin-producing *Escherichia coli* serotype O78:H^−^ in family, Finland, 2009. *Emerging Infectious Diseases*.

[19] Repetto HA (1997). Epidemic hemolytic-uremic syndrome in children. *Kidney International*.

[20] Sargison ND, Howie F, Mearns R, Penny CD, Foster G (2007). Shiga toxin-producing *Escherichia coli* as a perennial cause of abortion in a closed flock of Suffolk ewes. *Veterinary Record*.

[21] Rocha LB, Luz DE, Moraes CTP (2012). Interaction between Shiga toxin and monoclonal antibodies: binding characteristics and in vitro neutralizing abilities. *Toxins*.

[22] Bligh EG, Dyer WJ (1959). A rapid method of total lipid extraction and purification. *Canadian Journal of Biochemistry and Physiology*.

[23] Stritt A, Tschumi S, Kottanattu L (2013). Neonatal hemolytic uremic syndrome after mother-to-child transmission of a low-pathogenic stx2b harboring shiga toxin-producing *Escherichia coli*. *Clinical Infectious Diseases*.

[24] Burdet J, Zotta E, Franchi AM, Ibarra C (2009). Intraperitoneal administration of Shiga toxin type 2 in rats in the late stage of pregnancy produces premature delivery of dead fetuses. *Placenta*.

[25] Burdet J, Zotta E, Cella M, Franchi AM, Ibarra C (2010). Role of nitric oxide in Shiga toxin-2-induced premature delivery of dead fetuses in rats. *PLoS ONE*.

[26] Burdet J, Sacerdoti F, Cella M, Franchi AM, Ibarra C (2013). Role of TNF-*α* in the mechanisms responsible for preterm delivery induced by Stx2 in rats. *British Journal of Pharmacology*.

[27] Rasooly R, Do PM, Griffey SM, Vilches-Moure JG, Friedman M (2010). Ingested Shiga toxin 2 (Stx2) causes histopathological changes in kidney, spleen, and thymus tissues and mortality in mice. *Journal of Agricultural and Food Chemistry*.

[28] Yoshimura K, Fujii J, Tanimoto A, Yutsudo T, Kashimura M, Yoshida S-I (2000). Effects of Shiga toxin 2 on lethality, fetuses, delivery, and puerperal behavior in pregnant mice. *Infection and Immunity*.

[29] Zoja C, Angioletti S, Donadelli R (2002). Shiga toxin-2 triggers endothelial leukocyte adhesion and transmigration via NF-*κ*B dependent up-regulation of IL-8 and MCP-1. *Kidney International*.

[30] Odutayo A, Hladunewich M (2012). Obstetric nephrology: renal hemodynamic and metabolic physiology in normal pregnancy. *Clinical Journal of the American Society of Nephrology*.

[31] Hou S-A (2013). Woman with GN presenting during pregnancy. *Clinical Journal of the American Society of Nephrology*.

[32] Deng A, Baylis C (1995). Glomerular hemodynamic responses to pregnancy in rats with severe reduction of renal mass. *Kidney International*.

[33] Amaral MM, Sacerdoti F, Jancic C (2013). Action of Shiga toxin type-2 and subtilase cytotoxin on human microvascular endothelial cells. *PLoS ONE*.

[34] Soares MJ, Chakraborty D, Rumi MAK, Konno T, Renaud SJ (2012). Rat placentation: an experimental model for investigating the hemochorial maternal-fetal interface. *Placenta*.

